# Exploring fermentation with lactic acid bacteria as a pretreatment for enhancing antioxidant potential in broccoli stem powders

**DOI:** 10.3934/microbiol.2024013

**Published:** 2024-04-01

**Authors:** M Alegría Serna-Barrera, Claudia Bas-Bellver, Lucía Seguí, Noelia Betoret, Cristina Barrera

**Affiliations:** Instituto de Ingeniería de Alimentos-FoodUPV de la Universitat Politècnica de València, Camino de Vera s/n, 46022, Valencia, Spain

**Keywords:** broccoli stems, lactic acid fermentation, waste recovery, probiotics, antioxidants, freeze-drying, disruption intensity, powdered products

## Abstract

Fruit and vegetable industries face a major environmental challenge with food loss and waste. Broccoli stems, comprising 38% of the plant's total weight, are usually discarded by the industry producing fourth-range and ready-to-use products, despite being rich in antioxidants, vitamins, fiber, carotenoids, phenolic compounds, and glucosinolates. Addressing the challenge of reducing waste in this sector includes the production of stable and nutrient-concentrated powders, which can be consumed directly or used as ingredients in functional food formulation. This study investigated fermentation with lactic acid bacteria (*Limosilactobacillus reuteri*, *Lactiplantibacillus plantarum*, and *Lactobacillus salivarius*) as a pretreatment for enhancing antioxidant and probiotic potential in broccoli stem powders. Results showed maximum counts 24 h after inoculation, and no effect of the previous disruption intensity on microbial growth was observed. Fermenting broccoli stems for 24 h with the three microbial strains led to a significant increase in total phenols and flavonoids but to a general reduction in the samples' capacity to scavenge DPPH and ABTS free radicals. Overall, ground broccoli stems exhibited the most favorable antioxidant properties following the 24 h fermentation step. The subsequent freeze-drying and final grinding had minimal impact on the microbial population but significantly enhanced the extractability of the antioxidant compounds. This study offers a valuable reference for researchers and stakeholders exploring the development of new products and innovations from vegetable waste.

## 1. Introduction

Wasting food involves an inefficient use of the resources invested in its production, which are known to be limited. Consequently, it poses a significant global challenge, impacting ethical, social, environmental, and economic perspectives. The growing awareness of this issue aligns with the United Nations' formulation of the Sustainable Development Goals (SDGs) of 2015, which incorporates the target 12.3 that specifically addresses food waste. Efforts and initiatives implemented and developed since then have led to a 23% reduction in food waste occurring at the later stages of food's journey (household, food service, and retail level) in 2022 [1]. This progress marks the halfway point towards the goal of a 50% reduction in food waste across Europe's hospitality and food service sectors by 2025. However, as per FAO estimates [2], food losses occurring from production up to the retail level (including post-harvest on the farm, transport, storage, wholesale, and processing stages) have stayed at 13.2% since 2016. Among food groups, fruits and vegetables account for the highest percentage of food waste, comprising around 45% of the total amount entering the food supply chain; this is attributed to the larger proportion of inedible parts in these products and their higher perishability [3]. Addressing the challenge of reducing waste in this sector includes reusing strategies, which are limited to soil amendment and animal feed, as well as recycling strategies, based on the recovery of waste materials after a major modification of their characteristics [4]. The latter includes the production of stable and nutrient-concentrated powders, which can be consumed directly or used as ingredients in functional food formulation [5],[6].

One of the vegetables that generates remarkable amounts of waste is broccoli (*Brassica oleracea L*. var. *italica*). Consumption of this cruciferous plant in the last decade has witnessed a 10-fold increase, both in fresh and processed forms [7] due to a growing awareness of its health benefits supported by scientific studies. There are several epidemiological studies that identify an inverse correlation between broccoli consumption and the risk of suffering from diseases such as cancer, cardiovascular diseases, neurological diseases, or diabetes [8],[9]. Within the spectrum of bioactive compounds found in broccoli, isothiocyanates, glucosinolates, phenols (including flavonoids, anthocyanins, and phenolic acids), vitamins, and dietary fiber emerge prominently [10],[11]. However, as reported by Liu et al. [12], there is great variation in the nutrients and phytochemicals among the different botanical parts of broccoli. Florets exhibit elevated concentrations of amino acids, glucoraphanin, and neoglucobrassicin, while leaves have higher levels of carotenoids, chlorophylls, vitamins E and K, and the minerals Mn and Ca, as well as total phenolic content and DPPH antioxidant activity. In contrast, most sugars (fructose, glucose, sucrose, and maltose) are higher in the stems than in other tissues. At the industrial level, typically only the florets, constituting 15% of the total plant weight, are used. The prevalent forms of presentation include pre-cooked and deep-frozen options as well as ready-to-eat fourth-range products. Uses of broccoli stems are not limited to animal feed or compost, but also find application in pectin production. This type of pectin, characterized by its composition (75% galacturonic acid with a degree of methyl esterification of 56% and 1.1% acetyl), has the potential to be used as a thickener and emulsifier in food formulation [13].

Technological processing may lead to a variation in bioactive compounds. Based on the findings of Cieślik et al. [14], extending the blanching time from 3 to 10 min at 80 °C results in a reduction in total glucosinolates in broccoli, in terms of losses rising from 30% to 55.71%. Also, as observed by Severini et al. [15], hot water and steam blanching significantly decrease the ascorbic acid content from 151.32 mg/100 g dry weight in raw broccoli to 80.13 and 112.92 mg/100 g dry weight, respectively. Conversely, an increase in chemical extractability resulting from microwave blanching induces a considerable increase in the content of certain organic compounds, such as ascorbic acid and glucosinolates. Additionally, blanching of broccoli florets at lower temperatures (60 °C for 10–17 min) leads to increased myrosinase activity, thus facilitating bioconversion of glucoraphanin into sulforaphane, which is recognized as one of the most effective naturally occurring anticancer compounds in foods [16]. The activity of the myrosinase enzyme can also be triggered by the rupture of cell structures by cutting and grinding operations and with the participation of lactic acid bacteria in the fermentation process, as observed in the production of sauerkraut [17].

Such breakdown of cells by microbial enzymes can also result in the release of phenolic compounds covalently bound to them. This is the primary mechanism responsible for the boost in antioxidant capacity associated to fermentation of plant residues [18]. Fermenting microorganisms can also contribute to the production of compounds with antioxidant activity [19] and the transformation of some antioxidant compounds into others exhibiting higher activity [20]. In this line, Cai et al. [21] reported that the fermentation of broccoli florets puree with a co-culture of *Leuconostoc mesenteroides* (BF1, BF2) and *Lactobacillus plantarum* (B1, B2, B3, B4, B5) resulted in a 91% increase in the yield of sulforaphane and an 83% increase in total phenolic content [21]. This fermentation process also enhanced food safety by suppressing the growth of undesirable spoilage and pathogenic microorganisms, while improving sensory and physicochemical attributes. Fermentation by microorganisms recognized as probiotics in a 10^6^–10^7^ CFU/g concentration at the time of consumption can further enhance the nutritional value of the resulting product. Among probiotic microorganisms, *Limosilactobacillus reuteri* has the ability to prevent infections caused by pathogens and alleviate the effects of severe intestinal disorders, including colic in pediatric patients [22]. While this species is frequently used in dairy products' fermentation, several studies report good adaptation and survival in plant-derived products, such as cherry juice or broccoli puree [23]. On the other hand, *Lactiplantibacillus plantarum* is the predominant species in fermenting plant-derived food products due to its capacity to generate a substantial quantity of active enzymes, including amylases, β-glucosidases, decarboxylases, lactate dehydrogenases, peptidases, phenolic acid decarboxylases, phenol reductases, proteinases, and tannases [24]. Finally, the probiotic nature of *Lactobacillus salivarius* is attributed to its ability to survive in the stomach and small intestine, as well as to its capacity to adhere to and colonize the intestinal mucosa [25].

While various studies report the influence of processing and fermentation on the bioactive properties of cruciferous vegetables in terms of total polyphenol content, antioxidant capacity, glucosinolates, and degradation products [17],[26]–[28], there is limited information concerning the fermentation of broccoli stems and its effects on the physicochemical and nutritional properties of the fermented product. Therefore, the present study aims to investigate the ability of three lactic acid bacteria (*Limosilactobacillus reuteri*, *Lactiplantibacillus plantarum*, and *Lactobacillus salivarius*) to grow and biotransform the antioxidant compounds present in broccoli stems into forms with enhanced activity as affected by the degree of disruption (ground or chopped) of the plant tissue. The study also analyzes the additional impact of freeze-drying on both the viable count and the content of antioxidant compounds within the fermented broccoli stem. This investigation might provide a valuable resource for researchers and stakeholders interested in the functional composition of broccoli, thus contributing to the development of new products and innovations in the food industry.

## 2. Materials and methods

### 2.1. Plant material conditioning

The broccoli employed in this study originated from Murcia (Spain), yet it was procured from a local supermarket in Valencia (Spain). Until usage, the plant material was refrigerated at 4 °C. The initial step in sample preparation included separating the stems from the leaves and flowers. Stems were immersed for 5 min in a 200 ppm sodium hypochlorite aqueous solution at room temperature in a ratio of 2 g of sample to 10 mL of solution. After thoroughly rinsing with tap water, the stems were processed using a Thermomix® food processor (Vorwerk, Spain) at 10,000 rpm for 30 s (for ground samples, G) or at 10,000 rpm for 10 s (for chopped samples, C). Upon disruption, 100 g of plant material was placed in 370 mL sterile glass jars with twist-off closure. Pasteurization was achieved by immersing the jars in a water bath at 72 °C, ensuring that the center of the sanitized and disrupted broccoli stems stayed at the desired temperature for 1 min. Samples were allowed to cool to room temperature before being inoculated and fermented.

### 2.2. Bacterial cultures

The three lactobacilli strains used in the study (*Lactobacillus salivarius* spp. *salivarius* CECT 4063, *Lactiplantibacillus plantarum* CECT 749, and *Limosilactobacillus reuteri* CECT 925) were sourced from the Spanish Type Culture Collection (CECT, Valencia, Spain) based on their recognized potential for probiotic effects, proficiency in degrading and metabolizing various polysaccharides, and capacity to thrive in plant samples [29]. Following the guidelines provided by the supplier, the freeze-dried strains were reactivated in MRS broth and stored in vials at −20 °C with 15% (v/v) glycerol until needed. For inoculum preparation, 25 mL of MRS broth (Scharlau Chamie, Barcelona, Spain) was inoculated with the thawed content of a vial in a 250 mL Erlenmeyer flask and incubated at 37 °C overnight. The resulting 24-hours-old bacterial culture was used as the inoculum.

### 2.3. Experimental procedure

After tempering, the pasteurized material within glass jars underwent inoculation by the addition of 1 mL of inoculum. After gently stirring, the jars were sealed and placed in an oven for incubation at 37 °C. At 7, 24, 48, 72, and 96-h intervals, the jars were opened to monitor viable cell counts. Each microbial strain was inoculated into a total of four jars: two containing 100 g of ground broccoli stems, and the other two containing 100 g of chopped broccoli stems. After establishing the fermentation time that yielded the highest microbial concentration for each tested microorganism, it was implemented to ferment another batch of ground and chopped broccoli stems using the same experimental procedure in twist-off closure jars. This time, the fermented samples were intended for subsequent freeze-drying. To achieve this, the samples were subjected to freezing at −40 °C for 24 h in a CVN-40/105 freezer (Matek, Barcelona, Spain), followed by sublimation at −45 °C (condenser temperature) and 0.1 mbar for 48 h in a LyoQuest-55 laboratory freeze drier (Telstar, Terrasa, Spain). Then, the fermented and freeze-dried residue was converted into powder by grinding at 10,000 rpm for 20 s in a Thermomix® food processor (Vorwerk, Spain). Six powders in total were acquired, with two corresponding to each lactic acid bacteria and three associated with each pre-fermentation disruption intensity. An additional powder was obtained directly from the freeze-drying and grinding of fresh broccoli stems.

### 2.4. Analytical determinations

All the determinations described below were performed at least in triplicate. Regarding the fermented samples, measurements were duplicated for each of the two jars subjected to identical processing conditions.

Water activity (a_w_) of fresh broccoli stems was measured using an Aqualab® CX-2 dew point hygrometer from Decagon Devices Inc. (Pullman, WA, USA), with an accuracy of ±0.003 at a temperature of 25 °C.

pH of fresh and 24-h fermented broccoli stems was assessed using a S20 SevenEasy™ digital pH meter (Mettler-Toledo Inlab, Columbus, OH, USA), which was pre-calibrated using appropriate buffer solutions at pH 4 and 7.

Moisture (x_w_) of fresh broccoli stems was determined through a gravimetric method that involved measuring the weight loss of a known amount of sample during drying in a vacuum oven (Vaciotem, J.P. Selecta, S.A., Barcelona, Spain) at 60 °C and <133 mbar until a constant weight was achieved.

Total soluble solids (x_ss_) of fresh broccoli stems were determined using a thermostated Abbe refractometer NAR-3T (Atago, Tokyo, Japan) at 20 °C. The calculation involved the moisture content of the sample and the Brix degrees measured from the aqueous extract obtained by mixing the solid sample with water in a 1:10 (w/v) ratio.

Antioxidant properties were assessed on extracts obtained by combining fresh, fermented, or freeze-dried broccoli stem samples with an 80% (v/v) methanol/water solution at a ratio of 1:10 (w/v), stirring for 1 h in darkness using an ANC10 horizontal stirrer (Magna Equipments S. L., Barcelona, Spain) and subsequently centrifuging at 10,000 rpm for 5 min in a 5804/5804R centrifuge (Eppendorf Ibérica S.L.U., Madrid, Spain). Extract dilutions were performed as needed for subsequent analysis.

The total phenolic content was assessed using the Folin-Ciocalteu spectrophotometric method [30], which began by combining 0.125 mL of extract with 0.5 mL of bidistilled water and 0.125 mL of the Folin-Ciocalteu reagent (Scharlau Chamie, Barcelona, Spain). After a 6 min reaction in the dark, 1.25 mL of 7% (w/v) Na_2_CO_3_ solution and 1 mL of bidistilled water were added. The mixture was kept in the dark for 90 min, after which the absorbance was measured at 760 nm using a Helios Zeta UV/Vis spectrophotometer (Thermo Fisher Scientific, Waltham, MA, USA). A blank, with the sample replaced by 80% (v/v) methanol in bidistilled water, served as reference. Absorbance measurements (Abs) were compared with a standard curve of gallic acid (purity ≥98%, Sigma-Aldrich) within the concentration range of 0 to 0.4 g/L (x): Abs = 4.7377x + 0.0519 and R^2^ = 0.9968. The results were expressed as milligrams of gallic acid equivalents per gram of dry matter (mg GAE/g_db_).

The total flavonoid content was determined following the modified aluminum chloride (AlCl_3_) colorimetric method [31]. The process involved combining 1.5 mL of extract with 1.5 mL of a 2% (m/v) AlCl_3_ solution in methanol, initiating a reaction with the flavonoids that results in the development of a yellow color. The mixture was allowed to react for 10 min, followed by measuring the absorbance at 368 nm using a Helios Zeta UV/Vis spectrophotometer (Thermo Fisher Scientific, Waltham, MA, USA). A blank, with the sample replaced by 80% (v/v) methanol in bidistilled water, served as reference. Absorbance measurements (Abs) were compared with a standard curve of quercetin (purity ≥95%, Sigma-Aldrich) within the concentration range of 0 to 0.2 g/L (x): Abs = 10.493x − 0.0377 and R^2^ = 0.9938. The results were expressed as milligrams of quercetin equivalents per gram of dry matter (mg QE/g_db_).

The antioxidant activity was determined based on the samples' ability to scavenge DPPH (1,1-diphenyl-2-picrylhydrazyl) and ABTS [2,2′-azino-bis (3-ethylbenzothiazoline-6-sulfonic acid)] free radicals. DPPH method relies on assessing the change in absorbance, measured at 515 nm, within the purple DPPH solution as it interacts with the antioxidant compounds present in the sample [32]. In this process, 0.1 mL of extract and 2.9 mL of a 0.06 mM DPPH solution (Scharlau Chamie, Barcelona, Spain) in methanol were mixed in a spectrophotometric cuvette and allowed to react over a 2 h period before measuring the absorbance using a Helios Zeta UV/Vis spectrophotometer (Thermo Fisher Scientific, Waltham, MA, USA). A reference blank was established by substituting the sample with 80% (v/v) methanol in bidistilled water. The results were expressed as milligrams of trolox equivalents per gram of dry matter (mg TE/g_db_), derived from the percentage of inhibition (% INHIB) concerning the reference antioxidant trolox (purity ≥97%, Sigma-Aldrich) within the concentration range (x) of 0–0.4 g/L: % INHIB = 221.44x + 6.9758 and R^2^ = 0.9608. Following the procedure outlined by Re et al. [33], a 7 mM ABTS solution (Scharlau Chamie, Barcelona, Spain) in 2.45 mM potassium persulfate was left in darkness for 16 h at room temperature to generate the free radical. The solution was subsequently diluted in a phosphate buffer until an absorbance of 0.70 ± 0.02, as measured at 734 nm, was obtained. Afterward, 2.9 mL of this solution and 90 µL of extract were mixed in a spectrophotometer cuvette and allowed to react in the absence of light for 10 min before measuring the absorbance with a Helios Zeta UV/Vis spectrophotometer (Thermo Fisher Scientific, Waltham, MA, USA). A reference blank was created by replacing the extract with 80% (v/v) methanol in bidistilled water. A calibration line using trolox within the concentration range of 0 to 0.5 g/L enabled to express the results in milligrams of trolox equivalents per gram of dry sample (mg TE/g_db_): % INHIB = 166.51x + 0.6651 and R^2^ = 0.9945.

Viable cell count in MRS broth and in fermented broccoli stems, before and after freeze-drying, was determined by serial dilution using buffered peptone water (Scharlau Chamie, Barcelona, Spain), followed by plating on double-layer MRS agar (Scharlau Chamie, Barcelona, Spain) and incubation at 37 °C for 24–48 h. To create the initial dilution (10^−1^ g/mL) for solid fermented broccoli stems, 3 g of sample was blended at medium speed for 2 min with 27 mL of sterile peptone water in a stomacher bag.

### 2.5. Statistical analysis

Statistical analysis of the experimental data was conducted utilizing Statgraphics Centurion XVII software (Statpoint Technologies, Virginia, US). This involved applying both simple and multivariate analysis of variance (ANOVA) at a confidence level of 95% (p < 0.05).

## 3. Results

Table 1 displays the main physicochemical properties, including antioxidants, of broccoli stems. It also includes the correlation between the stems' weight and the total weight of the head of broccoli, given in percentage. As observed, the discarded part constituted 49% of the weight of the piece and its water content (x_w_) was 92% on average. Water activity (a_w_) of broccoli stems was 0.992 ± 0.004, creating an optimal environment for the proliferation of bacteria belonging to the *Lactobacillus* genus. In contrast, soluble solids content (x_ss_) of broccoli stems were quite low (3.03 ± 0.07%).

**Table 1.** Main physicochemical properties of fresh broccoli stems.

Property	Value
a_w_	0.992 ± 0.004
pH	6.9 ± 0.2
% stem	49 ± 4
x_w_ (g w/100 g)	92 ± 2
x_ss_ (g ss/100 g)	3.03 ± 0.07
total phenols (mg GAE/g_db_)	4.9 ± 0.6
total flavonoids (mg QE/g_db_)	0.33 ± 0.02
AOA_DPPH method (mg TE/g_db_)	2.6 ± 0.3
AOA_ABTS method (mg TE/g_db_)	2.0 ± 0.2

Mean ± standard deviation of three measurements

Regarding the antioxidant properties of fresh broccoli stems, the total content of phenolic compounds was similar to that obtained by Zhang & Hamauzu [34], Bandari & Kwak [35], and Bas-Bellver et al. [36], but 3.5-fold greater than that obtained by Liu et al. [12] for the same broccoli tissue. Among the phenolic compounds in the broccoli stems are flavonoids, with a total content lower than that reported by Bandari & Kwak [35] or Bas-Bellver et al. [36]. As for the antioxidant capacity measured by both the DPPH and the ABTS methods, it was lower than that reported by Saavedra-Leos et al. [7] in spray-dried broccoli stalk juice powders.

**Figure 1.**
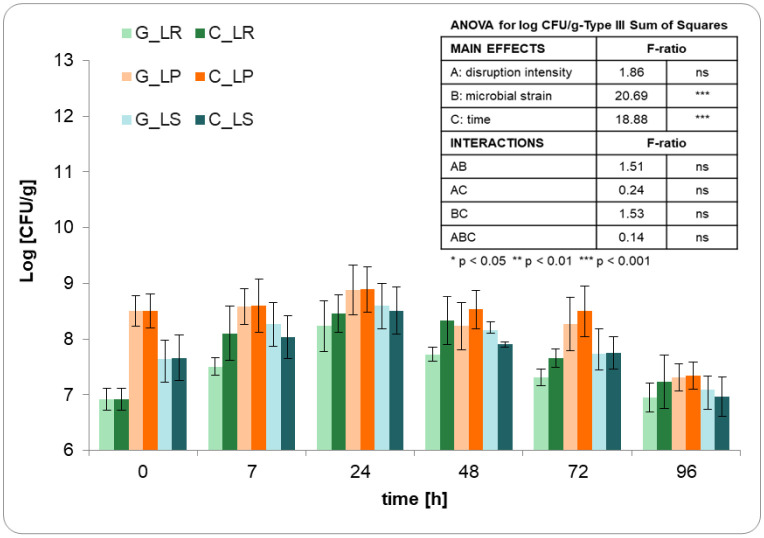
Microbial counts throughout the 4-day fermentation of ground (G) and chopped (C) broccoli stems with *Lactobacillus salivarius* (LS), *Lactiplantibacillus plantarum* (LP), and *Limosilactobacillus reuteri* (LR). Error bars represent the standard deviation of the means obtained from four measurements.

In Figure 1, the microbial counts obtained during the fermentation process of ground (G) and chopped (C) broccoli stems by *Lactobacillus salivarius* (LS), *Lactiplantibacillus plantarum* (LP), and *Limosilactobacillus reuteri* (LR) are shown. As evidenced, the initial viable counts ranged between 6.9 ± 0.3 log CFU/g for LR, 7.6 ± 0.4 log CFU/g for LS, and 8.6 ± 0.3 log CFU/g for LP, as a result of the microbial concentration reached during the inoculum preparation. These counts increased notably in the first few hours of fermentation, peaking after 24 h. Subsequently, the counts started to decline. Variation in cell counts over the fermentation time resembled a typical bacterial growth curve under restricted volume and nutrient conditions, encompassing four phases: lag, exponential, stationary, and death. In our case, the stationary phase was absent.

The ability of lactic acid bacteria to grow in broccoli stems (Table 2) correlated negatively with the initial microbial concentration. The ability of LP to grow in MRS medium was higher than that of LR and LS. Once inoculated, the capacity of lactic acid bacteria to grow in the broccoli medium correlated negatively with initial microbial concentration, as expected from the limited amount of substrate. In any case, the highest counts after 24 h of fermentation corresponded to LP. The degree of disruption prior to fermentation did not have a substantial impact on microorganism growth with the only exception of LR, in the first 7 h, which was significantly greater (p-value < 0.05) for chopped (C) than for ground (G) broccoli stems.

The decline in the microbial population observed after 24 h of fermentation (Table 2) was not determined by the strain type or the particle size of the substrate, leading to an average reduction of 1.4-log_10_. Since both LS and particularly LP exhibited greater mortality than growth over the 4-day fermentation period, the final counts in broccoli stems fermented with those strains were slightly lower than the initial ones. Throughout the entire fermentation, the microbial concentration consistently exceeded 10^6^–10^7^ CFU/g, which is the minimum threshold to consider a food product probiotic [37]. Indeed, consuming less than 10 g of broccoli stems fermented for 24 h with any of the three selected strains would meet the recommended daily intake of 10^8^–10^9^ CFU for beneficial health effects [29]. This amount increased to 150 g after 96 h from the start of fermentation, which remains a quite reasonable quantity for a daily portion.

**Table 2.** Logarithmic increase (values > 0) or decrease (values < 0) showing the *Lactobacillus salivarius* (LS), *Lactiplantibacillus plantarum* (LP), and *Limosilactobacillus reuteri* (LR) growth or demise from ground (G) or chopped (C) broccoli stems over a 4-day fermentation period.

*Treatment*	*Interval*				
	*0–7 h*	*0–24 h*	*24–48 h*	*24–72 h*	*24–96 h*
G_LS	0.6 ± 0.4a	1.0 ± 0.4bc	-0.4 ± 0.2a	-0.9 ± 0.5a	-1.5 ± 0.3b
C_LS	0.72 ± 0.14a	0.8 ± 0.4b	-0.61 ± 0.05a	-1.0 ± 0.2a	-1.5 ± 0.4ab
G_LP	0.38 ± 0.11a	0.39 ± 0.10a	-0.7 ± 0.4a	-0.7 ± 0.5a	-1.6 ± 0.2b
C_LP	0.55 ± 0.11a	0.6 ± 0.2ab	-0.66 ± 0.05a	-0.6 ± 0.3a	-1.5 ± 0.2b
G_LR	0.6 ± 0.2a	1.3 ± 0.5cd	-0.52 ± 0.13a	-0.94 ± 0.14a	-1.3 ± 0.3ab
C_LR	1.2 ± 0.5b	1.5 ± 0.3d	-0.51 ± 0.07a	-0.8 ± 0.2a	-1.2 ± 0.5a

a-d in the same column indicates statistically significant difference with a 95% confidence level (p-value < 0.05).

Mean ± standard deviation of four measurements.

**Figure 2.**
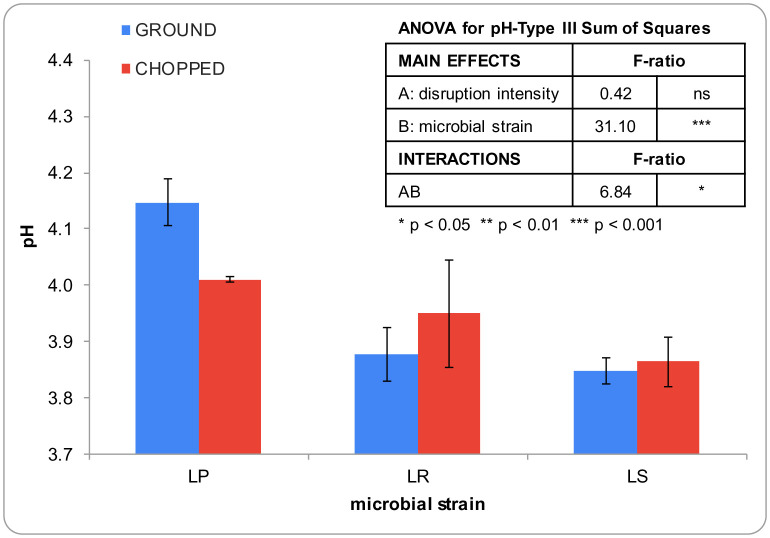
pH measurements of broccoli stems following a 24-h fermentation period with *Lactobacillus salivarius* (LS), *Lactiplantibacillus plantarum* (LP), and *Limosilactobacillus reuteri* (LR). Error bars represent the standard deviation of the means obtained from four measurements.

Figure 2 displays the pH measurements of broccoli stems following a 24-h fermentation period. The significant decrease observed in pH values (from 6.9 ± 0.2 to 3.95 ± 0.11) can be attributed to the formation of lactic, acetic, and propionic acids by lactic acid bacteria [38]. This fact is crucial for inhibiting the growth of microorganisms responsible for deterioration, thereby promoting the safety of the fermented food and extending its shelf life during subsequent storage [21]. Noticeably, the pH reduction was less evident when ground broccoli stems were fermented for 24 h with LP, in line with the reduced microbial growth observed under these conditions. Nevertheless, while the samples fermented with LR exhibited the highest microbial growth after 24 h, they did not exhibit the lowest pH values. Production of organic acids by lactic acid bacteria is not only dependent on the growth rate but also on the specific microorganism and strain. The impact of previous disruption intensity on pH decrease was uncertain. Grinding favored pH reduction in LR-fermented broccoli stems, while chopping favored it in LP-fermented samples. However, the pH decrease in LR-fermented samples remained consistent irrespective of the disruption intensity.

**Figure 3.**
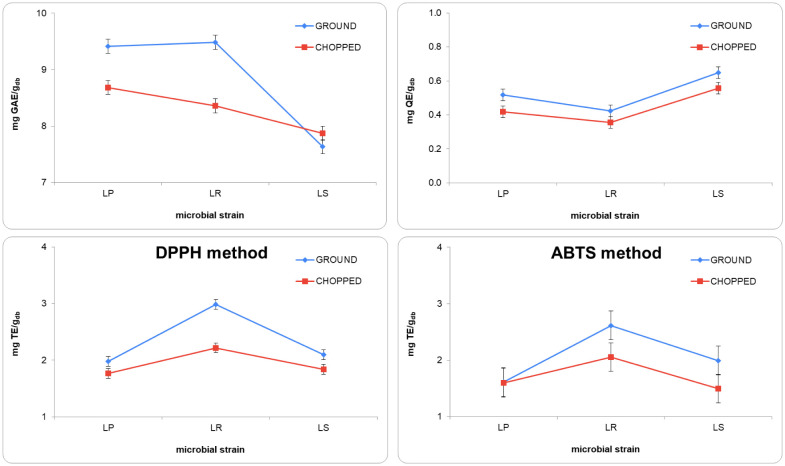
Interaction plot depicting the antioxidant properties of broccoli stems after a 24-h fermentation period with *Lactobacillus salivarius* (LS), *Lactiplantibacillus plantarum* (LP), and *Limosilactobacillus reuteri* (LR), influenced by the pre-fermentation disruption intensity. Error bars denote Fisher's least significant difference with a 95% confidence level (p-value < 0.05).

Figure 3 displays the antioxidant properties evaluated in broccoli stem, whether ground or chopped, after 24 h of fermentation. Based on the obtained results, fermenting broccoli stems for 24 h with the three microbial strains resulted in a notable rise in total phenols (between 1.57- and 1.95-fold higher), including flavonoids (between 1.12- and 1.95-fold higher). However, there was an overall decrease in the samples' ability to scavenge DPPH (between 0.67- and 0.84-fold lower) and ABTS (between 0.77-fold lower and unchanged) radicals, a trend that reversed only when ground broccoli stem was fermented for 24 h with LR. Grinding (G) generally implied a greater improvement in the antioxidant properties of fermented broccoli stems (p-value < 0.05) than chopping (C). Exceptionally, the total phenolic content in broccoli stems fermented for 24 h with LS was higher in C than in G samples. There were no significant differences in the antioxidant activity, as measured by the ABTS radical method, between C and G broccoli stems fermented for 24 h with LP. Differences between G and C samples regarding antioxidant properties became more significant when LR was used, with the exception of total flavonoid content. Antioxidant measurements by the DPPH and ABTS methods were significantly higher in samples fermented with LR, both for G and C broccoli stems. This was not in line with total phenols and total flavonoids results, the latter being lower for LR-fermented samples.

**Figure 4.**
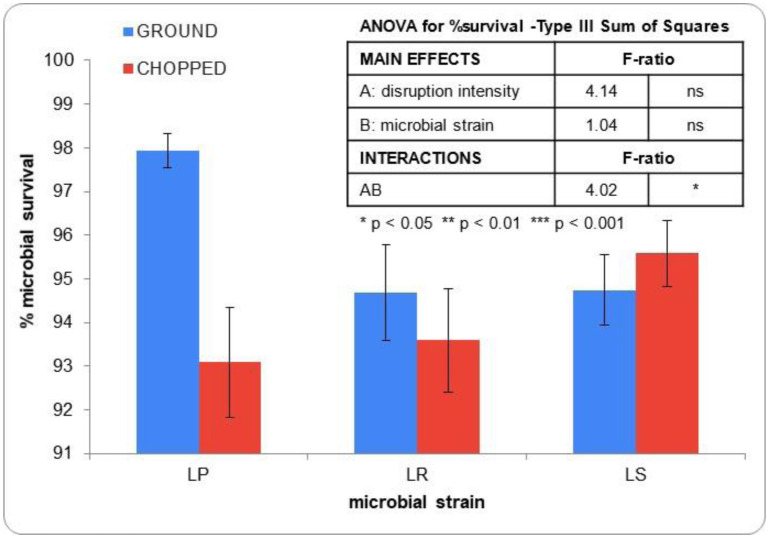
Survival to the freeze-drying step of the microbial population present in broccoli stems following a 24-h fermentation period with *Lactobacillus salivarius* (LS), *Lactiplantibacillus plantarum* (LP), and *Limosilactobacillus reuteri* (LR). Error bars indicate the standard deviation of the means obtained from four measurements.

In a further step, the 24-h fermented broccoli stems underwent freeze-drying to yield a stable powder suitable for incorporation into food formulations. Figure 4 depicts microbial survival to freeze-drying as affected by disruption intensity. Note that the survival of microbial population to freeze-drying was calculated from 24-h fermented broccoli stems, before and after freeze-drying, both expressed per gram of dry solid. In all cases, survival rates exceeded 90%, confirming that freeze-drying is an effective method for dehydrating probiotic foods and improving their stability without adversely impacting the viability of the beneficial microorganisms they contain. The statistical analysis of experimental data revealed that microbial survival was consistent (between 93.1% and 95.6%) and independent of previous disruption intensity. Grinding slightly promoted the survival of LP and LR to freeze-drying, an effect that was not observed for LS. In view of this result, antioxidant properties of freeze-dried products were assessed in powders obtained from ground samples.

Table 3 shows the antioxidant properties of freeze-dried ground broccoli stems after undergoing a 24-h fermentation process with the three potential probiotic strains. For reference, the antioxidant properties analyzed in the freeze-dried broccoli stems without fermentation (RAW) are also presented. To assess the impact of the dehydration stage on antioxidant properties, the percentage of variation (values in parentheses) was calculated from the concentrations in the sample before and after freeze-drying, both expressed on a dry basis.

Following the freeze-drying process, a notable enhancement was observed in all the antioxidant properties. An increase in the total phenols and flavonoid content was considerably more pronounced in non-fermented than in fermented broccoli stems, despite the fact that the content of total phenols and flavonoids was significantly higher in the fermented samples than in non-fermented ones. The microorganisms employed for fermentation did not have a significant impact on the total phenol content in the freeze-dried product. However, in terms of increase, it was notably higher in the samples that underwent fermentation with LS. Fermentation with LS also led to a broccoli stem powder exhibiting the highest total flavonoid content, which experienced the smallest increase during the freeze-drying step.

Differences between fermented and non-fermented samples in overall antioxidant activity were less pronounced, particularly when assessed in terms of the ability to scavenge DPPH free radicals. In contrast to the observations in fermented broccoli stems, powders exhibited antioxidant activities similar to or even higher than those obtained from unfermented broccoli stems (RAW broccoli). Powders derived from the fermentation of G broccoli stems with LS and LP for 24 h demonstrated the highest capacity to scavenge DPPH and ABTS radicals, respectively. This aligns with the fact that these samples exhibited a more substantial increase in their antioxidant activity during the freeze-drying process.

**Table 3.** Antioxidant properties of freeze-dried ground broccoli stems before and after undergoing a 24-h fermentation process with *Lactobacillus salivarius* (LS), *Lactiplantibacillus plantarum* (LP), and *Limosilactobacillus reuteri* (LR).

*Treatment*	*Antioxidant properties*			
	Total phenols (mg GAE/g_db_)	Total flavonoids (mg QE/g_db_)	AOA_DPPH (mg TE/g_db_)	AOA_ABTS (mg TE/g_db_)
G_LS	15.3 ± 0.2b (84%)	0.893 ± 0.003d (17%)	4.90 ± 0.02b (133%)	4.29 ± 0.03b (100%)
G_LP	15.2 ± 0.2b (63%)	0.787 ± 0.007b (52%)	4.0 ± 0.8a (100%)	4.6 ± 0.3c (185%)
G_LR	15.2 ± 0.2b (60%)	0.828 ± 0.011c (63%)	4.63 ± 0.05ab (32%)	4.33 ± 0.05bc (44%)
RAW	11.52 ± 0.08a (136%)	0.56 ± 0.04a (69%)	4.114 ± 0.014a (55%)	3.99 ± 0.03a (104%)

a-d in the same column indicates statistically significant difference with a 95% confidence level (p-value < 0.05).

Mean ± standard deviation of three measurements. The % of variation for each antioxidant property due to the freeze-drying is shown in parenthesis.

## 4. Discussion

Broccoli stems appear to be a good substrate for lactic acid bacteria growth, as deduced from the increase in viable cell counts observed within the first 24 h after inoculation without additional supplements. While each strain demonstrated different growth characteristics, all of them transitioned into the death phase within 24 h, probably as a result of the exhaustion of nutrients and the lactic acid production in the medium. In comparison to fermentation using autochthonous phyllosphere [39], inoculation with starter cultures reduced from 3 to 1 day the time needed to reach maximum lactic acid bacteria counts during the fermentation of broccoli stalks.

Among the three microorganisms tested, LR demonstrated the highest growth on the broccoli stem, whereas LP exhibited the lowest. These could be considered unexpected results, given that LP is among the most acid-tolerant species and is responsible for the second phase of fermentation of sauerkraut, so it is widely used as a commercial starter culture to minimize quality variation [40]. It could be argued that LR adapts more effectively than LS and LP to the composition of broccoli stems. These stems are characterized by a low content of soluble solids and a remarkable presence of antimicrobial compounds such as phenolics [41], isothiocyanates, and other glucosinolate hydrolyzates [42], as well as fatty acid derivatives [43]. Additionally, there are antinutritional compounds present, including oxalic acid, phytic acid, saponins, goitrogens, and trypsin inhibitors [44]. In fact, the capability of LR to thrive in plant matrices has been previously demonstrated by Mauro et al. [45], who developed a non-dairy probiotic drink based on cranberry juice and carrot. Yet, it is conceivable that the lower microbial concentration in the initial LR inoculum may have played a role in this outcome.

Regarding the intensity of the prior disruption of the plant material, it did not have a significant impact on the growth of any of the three strains tested, despite the expectation that the smaller particle size could enhance microorganisms' access to nutrients due to both nutrient release and increase of specific surface [46],[47]. Nonetheless, grinding could also have promoted a greater release of phenolic compounds and isothiocyanates with antimicrobial properties and, therefore, with a negative impact on microbial growth. However, grinding resulted in a more significant rise in the overall phenolic content, including those of the flavonoid type. This phenolic release would be the consequence of microbial enzymes that participate in the breakdown of vegetable walls, such as cellulases and glycosidases in lactic acid bacteria [39]. Microbial metabolism promotes the release of phenolic compounds but also the formation of new ones, a phenomenon that might be enhanced at smaller particle sizes, i.e., ground samples.

The positive correlation observed in earlier research between the phenolic transformation capacity of probiotic microorganisms and their growth cycle [48] could explain the considerably higher values in antioxidant activity and total phenols content obtained in samples fermented for 24 h with LR. Additionally, Filannino et al. [23] noted a strain-specific metabolism of polyphenols in broccoli puree. Interestingly, *L. reuteri* FUA3168 isolated from ting hydrolyzed chlorogenic acid into caffeic acid, which subsequently converted to dihydrocaffeic acid, a compound exhibiting higher bioavailability and antioxidant capacity compared with its precursors. Despite the increase in total phenols content, the slight decline observed in the antioxidant activity of the fermented samples can be attributed to a reduction in the content of other compounds with antioxidant properties, such as glucosinolates or vitamin C.

Freeze-drying the 24-h-fermented broccoli stems resulted in a minor reduction in the viability of the three strains tested. One of the main factors affecting the survival rate of lactobacilli during the freeze-drying process is the formation of large ice crystals, which have the potential to inflict severe damage to bacterial cells [49]. As deduced from the slightly higher survival rates found in ground broccoli stems, it seems that grinding over chopping may promote the formation of smaller ice crystals. While resistance to freeze-drying among different strains showed no significant differences, previous studies have demonstrated that *Lactobacillus plantarum* exhibits greater resistance than *Lactobacillus salivarius* and *Pediococcus acidilactici* to cell shrinkage and other damages during freeze-drying [50]. This would explain the higher survival rate of LP during the freeze-drying as compared with the other strains, in the case of the ground broccoli samples.

Freeze-drying is presented as a suitable technique for enhancing the content of compounds with antioxidant activity in broccoli stems. The observed rise can be attributed to disruption of the structural matrix induced by freeze-drying and subsequent grinding, thus facilitating the solvent extraction of antioxidant compounds. Freezing leads to the formation of ice crystals, which give rise to porous channels during subsequent sublimation. The porous structure obtained after freeze-drying is easy to grind and results in a fine powder that offers a large contact surface with the solvent used for extracting the compounds of interest, which, in turn, become more accessible [36]. Fermentation combined with freeze-drying implied an improvement of all the antioxidant properties analyzed with respect to non-fermented freeze-dried samples; however, the impact of freeze-drying, measured as the increase in the antioxidant attribute along drying, was sometimes higher for the non-fermented samples. Both freeze-drying and fermentation imply tissue disruption, which promotes the release of bioactive constituents and enhances extractability due to structural modifications. As observed by Bas-Bellver et al. [51], fermentation, driven by microbial enzyme activity, implies a more intense disruption of the tissue and yields finer powders after freeze-drying. Given that, as demonstrated earlier, structural changes induced by lactic acid fermentation significantly contributed to the release of phenols and flavonoids, subsequent freeze-drying and grinding played a comparatively minor role in the overall increase of their concentration. However, antioxidant compounds less positively affected by fermentation were more easily extracted from the finer powders resulting from freeze-drying and grinding.

## 5. Conclusions

In the present work, fermentation with lactic acid bacteria has been evidenced as a promising pretreatment for producing nutrient-enriched powders from broccoli residues. A 24-h fermentation period was chosen to maximize the presence of live cells of *Lactobacillus salivarius* (LS), *Lactiplantibacillus plantarum* (LP), and *Limosilactobacillus reuteri* (LR). This fermentation period led to a significant increase in the total phenols and flavonoid content of the plant material. Regarding the previous disruption's intensity, both grinding and chopping had comparable impact on the microbial counts. However, grinding yielded samples with statistically higher values (p-value < 0.05) for all the analyzed antioxidant properties. The fermented broccoli stems consistently exhibited counts exceeding 10^8^ CFU/g, remaining stable through subsequent freeze-drying and final grinding stages. The extraction of antioxidant compounds was further improved by freeze-drying and final grinding. Compounds that underwent a more substantial concentration increase due to microbial enzyme activity (total phenols and flavonoids) exhibited a lower rise in concentration following freeze-drying and grinding. Conversely, compounds that saw a decline in concentration following lactic acid fermentation exhibited a more pronounced increase after freeze-drying and grinding, presumably due to the finer powders obtained. The powdered product resulting from this research holds potential for reducing broccoli stem waste and serves as a promising ingredient in the development of innovative functional foods. Additional investigation into the survival of the added lactic acid bacteria during storage and in vitro simulated gastrointestinal digestion would be essential in this case to confirm the powders' potential probiotic effect.

## Use of AI tools declaration

The authors declare that Artificial Intelligence (AI) tools were not used in the creation of this article.
